# Exclusive intraoperative radiotherapy for invasive breast cancer in elderly patients (>70 years): proportion of eligible patients and local recurrence-free survival

**DOI:** 10.1186/s12893-016-0191-9

**Published:** 2016-11-15

**Authors:** Eric Lambaudie, Gilles Houvenaeghel, Amira Ziouèche, Sophie Knight, François Dravet, Jean Remy Garbay, Sylvie Giard, Hélène Charitansky, Monique Cohen, Christelle Faure, Delphine Hudry, Paul Azuar, Richard Villet, Pierre Gimbergues, Christine Tunon de Lara, Agnès Tallet, Marie Bannier, Mathieu Minsat, Michel Resbeut

**Affiliations:** 1 Institut Paoli Calmettes, 232 Boulevard de Sainte-Marguerite, 13009 Marseille, France; 2 CRCM, Marseille, France; 3 Institut René Gauducheau, Nantes, France; 4 Institut Gustave Roussy, Villejuif, France; 5 Centre Oscar Lambret, Lille, France; 6 Centre Claudius Regaud, Toulouse, France; 7 Centre Léon Bérard, Lyon, France; 8 Centre Georges François Leclerc, Dijon, France; 9 Hôpital de Grasse, Grasse, France; 10 Hôpital des Diaconesses, Paris, France; 11 Centre Jean Perrin, Clermont Ferrand, France; 12 Institut Bergonié, Bordeaux, France; 13 Aix Marseille Université, Marseille, France

**Keywords:** Breast cancer, Elderly patients, Intraoperative radiotherapy

## Abstract

**Background:**

To estimate the proportion of elderly patients (>70 years) with breast cancer eligible for an Exclusive IntraOperative RadioTherapy (E-IORT) and to evaluate their local recurrence-free survival rate.

**Methods:**

This retrospective study examining two cohorts focuses on patients over 70 years old: a multi-centric cohort of 1411 elderly patients and a mono-centric cohort of 592 elderly patients. All patients underwent conservative surgery followed by external radiotherapy for T0-T3 N0-N1 invasive breast cancer, between 1980 and 2008.

**Results:**

Within each cohort two groups were identified according to the inclusion criteria of the RIOP trial (R group) and TARGIT E study (T group). Each group was divided into two sub-groups, patients eligible (E) or non-eligible (nE) for IORT. The population of patients that were eligible in the TARGIT E study but not in the RIOP trial were also studied in both cohorts. The proportion of patients eligible for IORT was calculated, according to the eligibility criteria of each study. A comparison of the 5-year local or locoregional recurrence-free survival rate between eligible vs non-eligible patients was made.

In both cohorts, the proportion of patients eligible according to the RIOP trial’s eligibility criteria was 35.4 and 19.3%, and according to the TARGIT E study criteria was 60.9 and 45.3%.

The 5-year locoregional recurrence-free survival rate was not significantly different between RE and RnE groups, TE and TnE groups. In both cohorts RE and (TE-RE) groups were not significantly different.

**Conclusions:**

Our results encourage further necessary studies to define and to extend the eligibility criteria for per operative exclusive radiotherapy.

## Summary

The objective of this retrospective study was to estimate, in a cohort of 12,025 patients managed conventionally, the proportion of elderly patients (>70 years) eligible for exclusive intra-operative radiotherapy (IORT) and to evaluate local recurrence-free survival rates.

The results of this study suggest that up to 50% of patients presenting with a cN0, T0-2 tumor could be eligible for exclusive IORT with expected 7-year local recurrence-free survival rates ranging from 93 to 96%.

These results suggest the possible extension of the eligibility criteria for intra-operative exclusive radiotherapy.

## Background

Breast cancer has become the most frequent cancer in both “developed” and “developing” countries (http://globocan.iarc.fr/). Its management represents a major concern for public health at therapeutic and economic levels.

Moreover, robust publications have shown that in the case of conservative surgery for an invasive cancer, adjuvant whole-breast irradiation in association with a boost to the tumor bed significantly increases recurrence-free survival, without any difference compared to total mastectomy in terms of overall survival [1]. However, almost 90% of recurrences in the breast are located in the same quadrant [2].

Based on this clinical information, and with the aim of facilitating access to radiotherapy, partial breast irradiation techniques have been developed over the past 20 years; using brachytherapy, external radiotherapy and intraoperative radiotherapy.

Organized screening combined with these modern therapeutic approaches enable a less radical and invasive management of patients.

As suggested by the results of the Targit A trial, targeted intraoperative radiotherapy appears to be as safe as whole-breast external beam radiotherapy when the radiotherapy is delivered at the time of lumpectomy in selected patients [3]. The potential medico-economic impact must be considered in order to encourage the development of this technique as a second objective.

In France, the main objective of the RIOP trial [a French trial currently in progress conducted under the aegis of the National Cancer Institute (INCA - Institut National du Cancer) is medico-economic.

Concerning the elderly population, the future results of the TARGIT E trial [4] should give us a better knowledge of the indications for partial breast irradiation in this specific population.

Following our previously published study [5] concerning eligibility criteria in the general population of patients, the purpose of this new retrospective analysis focusing on patients over 70 years old, undergoing conservative treatment associating conservative surgery and conventional external radiotherapy for an invasive breast cancer was:

- to estimate the proportion of eligible patients for exclusive intraoperative radiotherapy (IORT) according to the criteria of the RIOP trial and the TARGIT E trial,
- to evaluate and compare using data from these two cohorts, the local and locoregional recurrence-free survival rates among patients who were both eligible and non-eligible for exclusive IORT according to the eligibility criteria of both trials mentioned above.

## Methods

**Fig. 1:**
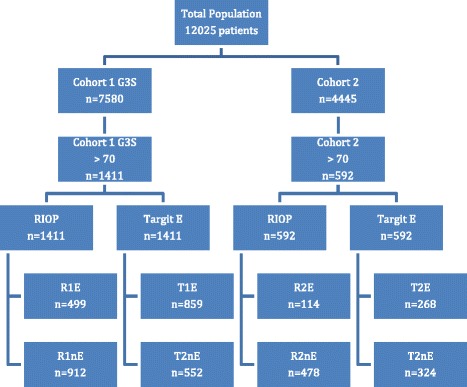
Flowchart figure. Keys: R = RIOP, T = Targit E, E = eligible, nE = not eligible

This retrospective study analyzed two different cohorts of patients who underwent conservative surgery followed by external radiotherapy for invasive, non-metastatic and non-inflammatory breast cancer (Fig. 1

).

- The first multi-centric cohort (cohort G3S) included 7580 patients treated in 13 centers between 1999 and 2008, all of which underwent a sentinel node biopsy followed or not by complementary axillary dissection for N0, T0 to T2 tumors.
- The second monocentric cohort (cohort 2) included 4445 patients treated at Paoli Calmettes Institute (IPC) between 1980 and 2005 with conservative surgery for tumors stage T0 to T3, N0 or N1 who underwent a sentinel node biopsy followed or not by complementary axillary dissection or alternatively immediate axillary dissection.

A first analysis concerning eligible patients within these cohorts was conducted and published recently using the same methodology [5]. For this task, we focused our study in both cohorts, on the subgroup of patients over 70 years old: 1411/7580 patients and 592/4445 patients for cohort 1 and cohort 2 respectively.

This study obtained ethics approval by our Institutional review board (Paoli Calmettes Institute’s ethical committee) was obtained and the data were used **confidentially** for research work.

### Anatomopathological information

The pathological analysis of each resected specimen defined tumor size, histological type, tumor stage, tumor grade according to the Scarff-Bloom-Richardson classification (SBR) [6], presence of lympho-vascular invasion and lymph node metastasis. Immunohistochemical techniques determined estrogen receptor (RE) and progesterone receptor (RP) status with a positive threshold of >10%. Data relating to the overexpression of the Her2 receptors was too recent in relation to the study period; therefor Her2 status was only defined for 857 patients (60.7%) and 89 patients (15%) over 70 years old, in the G3S cohort and the cohort 2 respectively.

### Adjuvant therapies

All patients were treated with 50 Gy whole-breast radiotherapy, most frequently associated with a 10–16 Gy boost to the tumor bed.

Adjuvant chemotherapy and a 5-year hormonotherapy in case of hormone-sensitive tumors were delivered according to institutional guidelines at the time of patient management. For all the patients with an established Her2 status, targeted therapy has been administrated when chemotherapy was indicated.

### Study groups and parameters studied

Two groups were identified within each cohort using the inclusion criteria of the RIOP and TARGIT-E trials. Each group was divided into two sub-groups, separating patients who were eligible for exclusive IORT from those who were not eligible: the RIOP trial with sub-groups R1E and R1nE for the G3S cohort, R2E and R2nE for the cohort 2 and the TARGIT E trial with the sub-groups T1E and T1nE for the G3S cohort, T2E and T2nE for the cohort 2. The sub-group of patients eligible in the TARGIT E study but not eligible in the RIOP study (TE-RE) were also analyzed in each cohort.

In each trial the eligibility criteria were based on preoperative clinical, radiological and histological data.

Patients’ eligibility (inclusion criteria) according to TARGIT E and RIOP trials are summarized in Table 1.

**Table 1:** Inclusion criteria for TARGIT E and RIOP trials

	TARGIT E	RIOP
Age (years)	≥70	≥55
Menopausal status	Required	Required
Unifocal tumor	Required	Required
Histology	Invasive ductal carcinoma	Invasive ductal carcinoma
Tumor size	≤35 mm	≤20 mm
No vascular invasion	Required	Required
SBR grade 1 and 2, hormone receptor positive, HER 2 negative	Not required	All of these criteria were required
No known BRCA mutation	Not required	Required

Patients eligible for the TARGIT E study were at least 70 years old and had a unifocal invasive ductal carcinoma. Three post-operative criteria indicated the requirement for additional external irradiation of the mammary gland: discrepancies concerning histologic type (invasive lobular carcinoma), the presence of an extensive intraductal carcinoma in situ and positive margins. The presence of lymph node involvement, lympho-vascular invasion, a tumor size above 35 mm and a proven multifocality or multicentricity could indicate an additional external irradiation. The results of this study are not yet available.

The eligibility criteria for an exclusive IORT according to the RIOP trial were: age ≥55 years, tumor size ≤ 2 cm, an unifocal invasive ductal carcinoma, tumor grade 1 or 2, positive hormone receptors (HR), the absence of lympho-vascular invasion and the absence of clinically involved lymph nodes. Her2 overexpression was considered an exclusion criterion. The presence of post-operative nodal invasion, of an extensive intraductal carcinoma in situ, positive excision margins and the failure to meet all the criteria stated above at the preoperative stage rendered complementary external radiotherapy necessary.

In our study, all the patients were menopausal and none of them have been referred for BRCA mutation’s detection.

For each cohort we calculated the proportion of patients eligible for exclusive IORT according to the criteria of each trial. We then compared, after conventional treatment combining breast conservative surgery and external radiotherapy, the 5-year and 7-year local recurrence-free survival rates of the following groups: RE groups versus the RnE groups, the TE groups versus the TnE groups and finally the RE groups versus (TE-RE).

All local recurrences have been confirmed by biopsy. Locoregional recurrences included axillary recurrences, supra and infraclavicular recurrences or intra-mammary recurrences. Concerning nodal recurrences, literature review indicates that it represents only a marginal number of locoregional recurrences; thus, recurrence-free survival can be considered as local recurrence-free survival in almost all cases [7–9].

This retrospective evaluation has limitations concerning clinical characteristics and follow-up; some criterions are missing in the description of each population.

### Statistical analysis

All statistical tests were two-sided. Comparisons were performed using the Chi-square test for percentages Student’s *t*-test for means (statistical significance was set at 0.05 for both tests) and log-rank test for survival (statistical significance set at 0.1). Statistical analyses were performed using SPSS version 16.0.

## Results

### Population characteristics

For the total population studied (12,025 patients for the two cohorts) the average age at diagnosis was 58 (median 58, range 22–101) in the G3S cohort and 55.6 years (median 55, range 20–91) in the IPC cohort. Within this population, all patients underwent conservative surgery and axillary lymph node staging (sentinel lymph node biopsy or complete axillary lymph node dissection). Adjuvant treatments were the following in the G3S and cohort 2 respectively: 36.7% (2784/7580) and 43.6% (1938/4445) patients received adjuvant chemotherapy and 84.3% (6373/7556) and 54.6% (2417/4422) patients were treated with adjuvant hormonal therapy. All patients received adjuvant radiotherapy with a whole-breast 50 Gy irradiation completed in most case with a 16 Gy boost to the tumor bed.

Regarding our study, patients over 70 years old represented 18.6% (1411/7580) of the G3S cohort 1 and 13.3% (592/4445) of the IPC cohort 2.

Characteristics of patients over 70 years old are shown in Table 2. There was a significant difference between both cohorts for the following characteristics: age, tumor size, hormonal receptor (HR) status, nodal status, grade, lympho-vascular invasion, and local recurrences with a population showing more unfavorable characteristics in the IPC cohort 2. These differences are confirmed when the eligible groups for RIOP (RE) and TARGIT E (TE) trials are considered, with fewer eligible patients in the IPC cohort 2 than in the G3S cohort 1.

**Table 2:** Characteristics of patients in the G3S and IPC cohorts, proportion of eligible patients for TARGIT E and RIOP trials

		G3S (*n* = 1411)	IPC (*n* = 592)	*p*
Age	70–79	1213 (86%)	488 (82.4%)	<0.05
	≥80	198 (14%)	104 (17.6%)	
pN	pN0	1113 (78.9%)	407 (76%)	0.05
	pN1	297 (21.1%)	143 (24%)	
Tumor type	Ductal	1064 (75.4%)	515 (87%)	<0.0001
	Lobular	177 (12.5%)	67 (11.3%)	
	Mixed	28 (2%)	9 (1.5%)	
	Other	137 (9.7%)	1 (0.2%)	
Tumor size	≤20 mm	1186 (84.6%)	402 (51.4%)	<0.0001
	>20 mm	216 (15.4%)	380 (48.6%)	
Grade	1	473 (33.9%)	196 (67.9%)	<0.02
	2	718 (51.5%)	271 (47.3%)	
	3	185 (13.3%)	106 (18.5%)	
	unknown	18 (1.3%)	19 (3.3%)	
Lymphovascular invasion	Yes	150 (13%)	154 (27.8%)	<0.0001
	No	1004 (87%)	399 (67.4%)	
Hormonal receptor	positive	1243 (90.5%)	405 (69%)	<0.0001
	negative	131 (9.5%)	182 (31%)	
Local recurrences	Yes	17 (1.2%)	18 (3%)	<0.01
	no	1394 (98.8%)	574 (97%)	
Eligible patients RIOP (RE)	Yes	499 (36.8%)	114 (19.3%)	<0.0001
	no	856 (63.2%)	478 (80.7%)	
Eligible patients TARGIT E (TE)	Yes	859 (60.9%)	268 (45.3%)	<0.0001
	no	552 (39.1%)	324 (54.7%)	

Table 3 presents in each cohort the eligible patients for the RIOP study (R1E and R2E) and the TARGIT-E study (T1E and T2E). Table 4 shows that in both cohorts eligible patients for RIOP trial had significantly different characteristics from the eligible patients in the TARGIT E trial and non eligible in the RIOP study (TE-RE).

**Table 3:** Characteristics of patients eligible for RIOP and TARGIT E trials of the G3S (R1E and T1E) and cohort 2 (R2E and T2E)

	COHORT G3S			COHORT 2	
	R1E	T1E		R2E	T2E
n	499/1411 (36.8%)	859/1411 (60.9%)		114/592 (19.3%)	268/592 (45.3%)
Age					
Median	73	73		74	74
Range	70–100	70–101		70–91	70–94
Tumor size mm					
Median (CI95 %)	10 (10.7–11.4)	11 (11.7–12.6)		12 (11.4–13.1)	15 (14.1–15.8)
Range	1–20	0,2–35		3–20	3–34
Histology					
Ductal	423 (84.8%)	748 (87.5%)		114 (100%)	268 (100%)
Lobular	0	0		0	0
Mixed	0	0		0	0
Other	76 (15.2%)	107 (12.5%)		0	0
Grade					
1	268 (53.7%)	358 (42.3%)	1–2	114 (100%)	229 (88.1%)
2	231 (46.3%)	367 (43.3%)	3	0	31 (11.9%)
3	0	109 (12.9%)			
Unknown	0	0			
pN					
pN0	499 (100%)	858 (100%)	pN0	114 (100%)	243 (90.7%)
i+	0	0	≤3	0	0
mic	0	0	>3	0	0
macro	0	0	unknown	0	25 (9.3%)
HR					
+	499 (100%)	745 (89,4%)		114 (100%)	175 (65,5%)
-	0	88 (10,6%)		0	92 (34,5%)
Her2 amplification					
Yes	13 (3.7%)	31 (5.6%)		2 (7.4%)	8 (17.8%)
No	337 (96.3%)	525 (94.4%)		25 (92.6%)	37 (82.2%)
Lymphovascular invasion					
Yes	0	0		0	0
No	499 (100%)	673 (100%)		114 (100%)	243 (100%)
Margins					
Positive	0	0		0	0
Negative	499 (100%)	859 (100%)		114 (100%)	268 (100%)
Multifocality					
Yes	0	0		0	0
No	499 (100%)	859 (100%)		114 (100%)	268 (100%)
Chemotherapy					
Yes	11 (2.4%)	54 (6.9%)		11 (9.6%)	39 (14.6%)
No	440 (97.6%)	724 (93.1%)		103 (90.4%)	229 (85.4%)
Hormonotherapy					
Yes	428 (86.7%)	670 (78%)		114 (100%)	175 (65.5%)
No	69 (13.9%)	189 (22%)		0	92 (34.5%)

**Table 4:** Comparison of patients of the G3S cohort 1 (R1E) and IPC cohort 2 (R2E) eligible for the RIOP trial with patients eligible for the TARGIT E study and not eligible for RIOP (TE-RE)

	G3S COHORT 1		*p*		IPC COHORT 2		*p*
	R1E	T1E-R1E			R2E	T2E-R2E	
n	499/1411 (36.8%)	304/803			114/592 (19.3%)	154/592 (26%)	
Age							
Median	73	74	0.376		74	74	0.27
Range	70–100	70–101			70–91	70–94	
Tumor size mm							
Median (CI95 %)	10 (10.7–11.4)	13 (13.7–15.4)	<0.0001		12 (11.4–13.1)	16.5 (15.7–18.1)	0.001
Range	1–20	0,2–35			3–20	3–34	
Histology							
Ductal	423 (84.8%)	280 (92.4%)	0.001		114 (100%)	154 (100%)	NS
Lobular	0	0			0	0	
Mixed	0	0			0	0	
Other	76 (15,2%)	23 (7,6)			0	0	
Grade							
1	268 (53.7%)	63 (20.8%)	<0.0001	1–2	114 (100%)	115 (78.8%)	<0.0001
2	231 (46.3%)	118 (38.9%)					
3	0	109 (36%)		3	0	31 (21.2%)	
Unknown	0	13 (4.3%)					
pN							
pN0	499 (100%)	304 (100%)	NS	pN0	114 (100%)	129 (100%)	NS
i+	0			≤3	0	0	
mic	0			>3	0	0	
macro	0			unknown	0	0	
HR							
+	499 (100%)	211 (70.6%)	<0,0001		114 (100%)	61 (39.9%)	<0.0001
-	0	88 (29.4%)				92 (60.1%)	
Her2 amplification							
Yes	13 (3.7%)	15 (8.8%)	0.015		2 (7.4%)	7 (9.9%)	0.08
No	337 (96.3%)	155 (91.2%)			25 (92.6%)	64 (90.1%)	
Lymphovascular invasion							
Yes	0	0	NS		0	0	NS
No	499 (100%)	159 (100%)			114 (100%)	129 (100%)	
Unknown							
Margins							
Positive	0	0	NS		0	0	NS
Negative	499 (100%)	304 (100%)			114 (100%)	146 (100%)	
Multifocality							
Yes	0	0	NS		0	0	NS
No	499 (100%)	304 (100%)			114 (100%)	151 (100%)	
Chemotherapy							
Yes	11 (2.4%)	42 (14.5%)	<0.001		11 (9.6%)	28 (18.2%)	0.036
No	440 (97.6%)	248 (85.5%)			103 (90.4%)	126 (81.8%)	
Hormonotherapy							
Yes	428 (86.7%)	206 (68%)	<0.0001		114 (100%)	61 (39.9%)	<0.0001
No	69 (13.9%)	97 (32%)			0	92 (60.1%)	
Radiotherapy boost to tumor bed							
Yes					92 (80.7%)	116 (84.7%)	0.25
No					22 (19.3%)	21 (15.3%)	

*NS* no significative difference

### Proportion of eligible patients and locoregional recurrence-free survival

In the G3S cohort the proportion of patients over 70 years old eligible for exclusive IORT was 35.4% (499/1411) and 60.9% (859/1411) when using eligibility criteria of the RIOP and TARGIT E studies respectively. 5-year and 7-year locoregional recurrence-free survival rates were 94.2 and 94.2% respectively for the R1E group versus 93.7 and 89% for the R1nE group; 94.1 and 92.3% for the T1E group versus 93.4 and 89.8% for the T1nE group. Locoregional recurrence-free survival was not significantly different between the T1E and T1nE (log rank = 0,266) groups and between the R1E and R1nE (log rank = 0,136) groups.

We did not observe any significant difference between the R1E and (T1E-R1E), log rank = 0,210 (Table 5).

**Table 5:** Loco regional recurrence-free survival in the G3S cohort 1

		R1	R1nE	T1	T1nE	R1E vs (T1E-R1E)
	Number	496	853	856	549	304
5 years	%	94.2	93.7	94.1	93.4	94.3
	Standard error	0.012	0.01	0.010	0.012	0.016
	Exposed to the risk	244	348	377	247	100
7 years	%	94.2	89	92.3	89.8	86.4
	Standard error	0.012	0.019	0.015	0.019	0.047
	Exposed to the risk	69	81	91	63	17
Log Rank			0.136		0.266	0.210

In the entire G3S cohort overexpression of Her2 was not related to a higher rate of locoregional recurrences log rank = 0.894.

The study of the sub-populations showed that the eligible patients both in the TARGIT-E and the RIOP studies with tumors overexpressing Her2 did not present locoregional recurrence significantly more frequently than patients without overexpression of Her2. In the IPC cohort the proportion of patients over 70 years old eligible for exclusive IORT was 19.3% (114/592) and 45.3% (268/592) when using eligibility criteria of the RIOP and TARGIT E trials respectively. The 5-year and 7-year local recurrence-free survival rates were 98.6 and 98.6% respectively for the R2E group versus 97 and 96.6% for the R2nE group; 97.7 and 97.7% for the T2E group versus 97 and 96.3% for the T2nE group. There was no significant difference in local recurrence-free survival between the T2E and T2nE groups (log rank = 0.319) and between the R2E and R2nE groups, (log rank = 0.314). Nor was there a significant difference in local recurrence-free survival between the R2E and T2E-R2E groups (log rank = 0.322), (Table 6).

**Table 6:** Loco regional recurrence-free survival in the IPC cohort 2

		R2	R2nE	T2	T2nE	R2E vs (T2E-R2E)
	Number	114	478	268	324	315
5 years	%	98.6	97	97.7	97	96.2
	Standard error	0.014	0.009	0.012	0.011	0.013
	Exposed to the risk	31	235	114	152	143
7 years	%	98.6	96.6	97.7	96.3	96.2
	Standard error	0.014	0.010	0.012	0.012	0.013
	Exposed to the risk	9	129	59	79	79
Log Rank			0.314		0.319	0.322

## Discussion

Although conservative surgery followed by post-operative adjuvant radiotherapy, associating whole-breast irradiation followed by a boost to the tumor bed is considered the standard care of early stage breast cancer [1], a current aim is to reduce treatment duration and associated costs. Though this option could benefit the entire population of patients treated for breast cancer, elderly patients, who are generally more fragile, represent an excellent target population. For this reason partial and accelerated breast irradiation techniques by means of brachytherapy, external radiotherapy and intra-operative radiotherapy have been developed.

Certain advantages of these techniques should be underlined: such as radiobiological benefits enabling a reduction in the time lapse between surgery and radiotherapy and an increase in the dose of equivalent irradiation.

Additionally, irradiation techniques such as IORT delivered during the surgical procedure also seem to act favorably on the tumor microenvironment [2].

For selected patients, these techniques enable a reduction in the volume treated and the duration of the irradiation without a reduction in survival. In a recent non-inferiority phase 3 randomized trial, Vaidya et al. reported no significant difference in terms of local recurrence with a 4 year follow-up in 2232 randomized patients between external radiotherapy and intra-operative radiotherapy with 50kv type intrabeam photons [3]. The updating of this series has confirmed the efficacy of this treatment over 5 years for patients who received IORT at the time of the initial lumpectomy (pre pathology group), and not after definitive pathology during a second surgical time (post pathology group) [10].

Other techniques of accelerated partial breast irradiation using brachytherapy, external radiotherapy of the ELIOT system have been studied, confirming the safety of this type of irradiation [11–13]. The difference in the proportion of eligible patients for exclusive IORT observed between the G3S and IPC cohorts for elderly patients (35.4 and 19.3% eligibility according to the criteria of the RIOP trial; 60.9 and 45.3% according to the TARGIT E trial) can be explained by the significantly greater number of patients presenting with unfavorable tumor characteristics in the IPC cohort. In the G3S cohort, the 5-year locoregional recurrence-free survival rate was 94.2 and 94.1% for patients eligible for IORT according to the criteria of the RIOP and TARGIT-E trials respectively and in the IPC cohort of 98.6 and 97.7% respectively for the both groups. We therefore expect comparable local recurrence rates if patients are effectively treated by exclusive IORT.

The eligibility criteria for the TARGIT-E trial were less restrictive than those of the RIOP trial; for this reason we compared local and locoregional recurrence-free survival rates of the group of patients eligible for the RIOP trial with the patients eligible for the TARGIT-E trial but not eligible for the RIOP trial (RE versus TE-RE): in both cohorts we observed no significant difference between these groups in terms of local or locoregional recurrence-free survival rate. These results therefore encourage us to extend the eligibility criteria for exclusive IORT, which were very restrictive in the RIOP trial. In order to confirm this state of the art, we are waiting for the results of the TARGIT E trial. In this study preoperative breast MRI is not required for inclusion; nevertheless this raises the question of its value for disqualifying patients with bifocal or multifocal tumors as in the RIOP trial.

In order to extend the indications for IORT, the overexpression of Her2 has been screened to confirm or not its negative impact on survival, as it has been considered as an exclusion criteria; its value as an independent risk factor compared to other criterions (age, emboli, and hormonal receptors status) is under discussion [14, 15]. In our study this factor does not compound the risk of local recurrence among eligible patients and, consequently, may not be considered as an exclusion factor if a treatment by exclusive IORT is planned.

Although local recurrence represents the main risk for patients who are eligible for partial breast radiotherapy, the team’s decision (surgeon/radiotherapist) could be guided by different tools that are still in development. In a study reported by Sanghani et al., significant risk factors for local recurrence were identified by developing a model with patients treated in nine randomized trials. The most significant factors were age < = 40 years (HR 2.03), positive margins (HR 2.19), positive HRs (HR 0.73) and grade III tumors (HR 1.55) [16]. In a metaanalysis, Van Nes et al. developed a prognostic index to predict the risk of local recurrence by identifying three risk groups. However, this study compares local recurrence after conservative surgery and after mastectomy and does not take into account major factors such as the SBR grade, the HR status or lympho-vascular invasion [17].

Finally, the potential economic impact of this technique is undeniable. Intra-operative radiotherapy currently provides the best option to reduce hospital stay and offers the possibility to perform one-day treatment. This advantage is particularly interesting in elderly patients, who usually present more comorbidity, in order to limit the impact of the treatment. In our study and according to the sub-groups of elderly patients (≥70 years), the outermost percentages of patients who could have benefited from exclusive IORT were 19.3 and 60.9%: These are so many patients in whom external radiotherapy could be avoided. The savings entailed by such a procedure have been estimated: in the United Kingdom this treatment would shorten the waiting lists for post-operative radiotherapy, saving an estimated 23 million dollars. In the USA, IPAS has already been proposed as an alternative to standard irradiation among patients of the most favorable ASTRO group in order to reduce time and costs of journeys for post-operative external radiotherapy [18]. ESTRO also provided a classification to guide the selection of patients suitable for partial accelerated irradiation of the breast [19]. A recent study of a cohort of 59,396 patients has shown an increase in the use of partial accelerated irradiation of the breast from 3.4% in 2003 to 12.8% (*p* < 0.001) in 2008 [20]. On this matter, the RIOP trial is ongoing in France to study the economic impact of this technique in our country.

The next step if we want to decrease cost and morbidity in relation with adjuvant treatment and particularly external beam radiation therapy, is to select a subset of patients who did not benefit of breast radiation after lumpectomy. This is aim of the CALGB 9343 trial where patients older than 70 years old with a clinical stage 1 breast cancer ER positive, have been randomised between adjuvant radiation therapy followed by tamoxifene (Tam RT group *n* = 317) or tamoxifene alone (Tam group *n* = 319). Hughes et al. [21] has reported the results of this study, with no difference in term of overall survival but a significantly longer time to local recurrence in the TamRT group (observed HR, 0.18; 95% CI, 0.07 to 0.42; *P*.001). Long-term follow-up of CALGB 9343 confirms and extends that in women age >70 years with clinical stage I, ER-positive breast cancer treated with lumpectomy followed by tamoxifen, irradiation adds no significant benefit in terms of survival: the place of exclusive IORT has to be discuss to reduce locoregional recurrence in this low risk population. But to date, external beam radiotherapy after lumpectomy remains the standard of care in Europe, even in case of elderly patients with a low risk of locoregional recurrence.

## Conclusion

Recent data from the TARGIT A trial are encouraging in terms of effectiveness, safety and toxicity, although a more extensive follow-up is necessary. Considering IORT’s potential advantages in terms of cost/efficiency, our study shows that its potential is underestimated.

The selection of patients remains an important issue but, given the frequency of breast cancer, a significant number of treatments could be carried out in this way. Our results suggest that when considering RIOP or TARGIT E’s inclusions criterions, 19.3 to 60.9% T0-2 N0 patients could be eligible for exclusive IORT even in case of Her2 overexpression, with no difference concerning recurrence-free survival compared to non-eligible patients.

If intra operative radiotherapy must be done at the time of surgery as demonstrated in the TARGIT A trial, our results encourage further necessary studies to define and to extend the eligibility criteria for per operative exclusive radiotherapy.

## Abbreviations

ASTRO: American Society for Radiotherapy & Oncology
E: Eligible
ESTRO: European Society for Radiotherapy & Oncology
Her2: Human Epidermal Growth Factor Receptor-2
HR: Hormone receptor
IORT: Intra-operative radiotherapy
IPC: Paoli Calmettes Institute
MRI: Magnetic resonance imaging
nE: Non-eligible
RE: Estrogen receptor
RP: Progesterone receptor
SBR: Scarff-Bloom-Richardson classification
T group: TARGIT E study
